# TAFRO Syndrome in Caucasians: A Case Report and Review of the Literature

**DOI:** 10.3389/fmed.2017.00149

**Published:** 2017-09-22

**Authors:** Céline Louis, Sandrine Vijgen, Kaveh Samii, Yves Chalandon, Louis Terriou, David Launay, David C. Fajgenbaum, Jörg D. Seebach, Yannick D. Muller

**Affiliations:** ^1^Division of Hematology, Geneva University Hospital, University of Geneva, Geneva, Switzerland; ^2^Department of Pathology, Geneva University Hospital, University of Geneva, Geneva, Switzerland; ^3^Department of Internal Medicine and Clinical Immunology CHU, University of Lille, U995, Lille Inflammation Research International Center, INSERM, Centre national de référence maladies systémiques et auto-immunes rares (sclérodermie systémique), Lille, France; ^4^Division of Translational Medicine and Human Genetics, Perelman School of Medicine, University of Pennsylvania, Philadelphia, PA, United States; ^5^Division of Clinical Immunology and Allergy, Geneva University Hospital, University of Geneva, Geneva, Switzerland

**Keywords:** TAFRO, Caucasian, review of literature, Castleman–Kojima disease, multicentric Castleman’s disease

## Abstract

**Background:**

TAFRO syndrome has been reported in Japan among human herpesvirus 8 (HHV-8)-negative/idiopathic multicentric Castleman’s disease (iMCD) patients. To date, the majority of iMCD patients with TAFRO syndrome originate from Japan.

**Case presentation:**

Herein, we report a 67-year-old HIV/HHV-8-negative Caucasian iMCD patient diagnosed with TAFRO. He presented with marked systemic inflammation, bicytopenia, terminal renal insufficiency, diffuse lymphadenopathies, and anasarca. Lymph node and bone marrow biopsies revealed atrophic germinal centers variably hyalinized and megakaryocytic hyperplasia with mild myelofibrosis. Several other biopsies performed in kidneys, liver, gastrointestinal tract, prostate, and lungs revealed unspecific chronic inflammation. The patient had a complete response to corticosteroids, tocilizumab, and rituximab. He relapsed twice following discontinuation of rituximab. When reviewing the literature, we found seven other Caucasian cases with TAFRO syndrome. There were no significant differences with those described by the Japanese cohort except for the higher frequency of kidney failure and auto-antibodies in Western patients.

**Conclusion:**

This case illustrates that patients with TAFRO syndrome can develop non-specific inflammation in several tissue sites. Furthermore, this case and our review of the literature demonstrate that TAFRO syndrome can affect Caucasian and Japanese patients highlighting the importance of evaluating for this syndrome independently of ethnic background.

## Background

Multicentric Castleman’s disease (MCD) is diagnosed clinicopathologically (1). Human herpesvirus 8 (HHV-8), a gamma herpesvirus first identified in Kaposi’s sarcoma, is the etiological cause of MCD in individuals who are HIV-positive or immunocompromised for another reason (2). In HHV-8-associated MCD, HHV-8 infects lymphocytes, macrophages, and endothelial and epithelial cells, lytically replicates in immunocompromised individuals, and signals for production of a viral homolog of human IL-6, which induces a cytokine storm and atypical lymphoproliferation (3). HHV-8-negative MCD patients in whom the etiology is not known are referred to as idiopathic multicentric Castleman’s disease (iMCD). A large study of Japanese MCD patients found that HHV-8-associated MCD occurs at a lower frequency than in European cohorts (4). None of the 79 Japanese HIV-negative MCD cases were HHV-8-positive whereas to 7/17 cases in a French cohort (2) and 6/14 cases in an Italian cohort (5) were HHV-8-positive (4). At the time, these controversial data were postulated to be due to the raciogeographical difference and the low prevalence of HHV-8 seropositivity in healthy Japanese individuals, although this hypothesis has never been confirmed/disproved (4).

In 2008, Kojima et al. proposed the first clinical sub-classification of HHV-8-negative/iMCD including idiopathic plasmacytic lymphadenopathy (IPL)-type and non-IPL type. IPL type exhibits marked hyperimmunoglobulinemia, severe inflammation, and thrombocytosis with follicular hyperplasia and interfollicular sheets of mature plasmatic cells. Non-IPL type exhibits anasarca, inflammation, and thrombocytopenia with atrophic lymphoid follicules and a hyaline vascular (HV)/mixed pattern of the germinal center (GC) (6). The clinicopathological description of non-IPL-iMCD corresponds very closely to the recently identified TAFRO syndrome or Castleman–Kojima disease described first by Takai et al. (7, 8). Since then, several cases have been published, and formal diagnostic criteria were established in 2015 based on 28 patients all originating from Japan (9). Major required criteria for TAFRO syndrome include (I) anasarca, (II) thrombocytopenia (<100 G/l), and (III) systemic inflammation. Two of the following four minor criteria are also required: (I) Castleman’s disease-like features on lymph node biopsy, (II) reticulin myelofibrosis and/or hyperplasia of megakaryocytes in the bone marrow, (III) mild organomegaly of the lymphoid organs and the liver, and (IV) progressive renal insufficiency. Malignancies (including POEMS), auto-immune disorders (including IgG4-related disease), infectious diseases, and auto-immune thrombocytopenia should be excluded before the diagnosis of TAFRO is made (9). Currently, it remains controversial as to whether TAFRO syndrome is a distinct entity from iMCD, a clinical subtype of iMCD, or a syndrome with multiple overlapping diseases (10).

We report the complex management of a Caucasian iMCD patient with TAFRO syndrome and identified seven other cases of Caucasian patients in the literature. The clinicopathological findings of this series of eight cases was systematically analyzed and compared with those described in Japan.

## Case Presentation

### Clinical Presentation

Herein, we report on a 67-year-old Caucasian originating from Portugal admitted to the hospital for fever of unknown origin and asthenia. His medical history was relevant for hypertension, insulin-dependent type two diabetes, and vitiligo and was on aspirin cardio, lisinopril, torasemid, omeprazole, and insulin therapy. On clinical examination, we noticed anasarca with pleural effusion, ascites, and edema of the lower limbs. Laboratory tests revealed microcytic anemia (hemoglobin 100 g/l, MCV 75 fl) with thrombocytopenia (73 G/l), marked elevation of C-reactive protein (204 mg/l), renal insufficiency (creatinine 463 µmol/l), and cholestasis (alkaline phosphatase 300 U/l, gamma-GT 56 U/l), with normal transaminases. Urinalysis showed severe proteinuria (5.29 g/l) and glomerular microhematuria. Immunological evaluation revealed normal IgG, IgM, IgA, and complement (C3, C4) levels, anti-nuclear antibodies (1/640 speckled), anti-SSA, anti-actine, and anti-parietal cell antibodies were positive. The infectious work-up was negative (HIV, hepatitis B/C, CMV, TB spot, and blood culture) except for EBV that was slightly positive by PCR (3,560 copies/ml). A thoraco-abdominal CT scan detected multiple mediastinal, axillary and retroperitoneal adenopathies, pleural and pericardial effusions, hepatosplenomegaly, and ascites. There was no sign of peripheral hemolysis (absence of schsitocytes). Finally, a cytokine profile showed elevation of IL-6, VEGF, soluble IL-2 receptor, and TNF-α, whereas IL-8 was normal.

### Diagnostic Course and Biopsies

**Figure 1:**
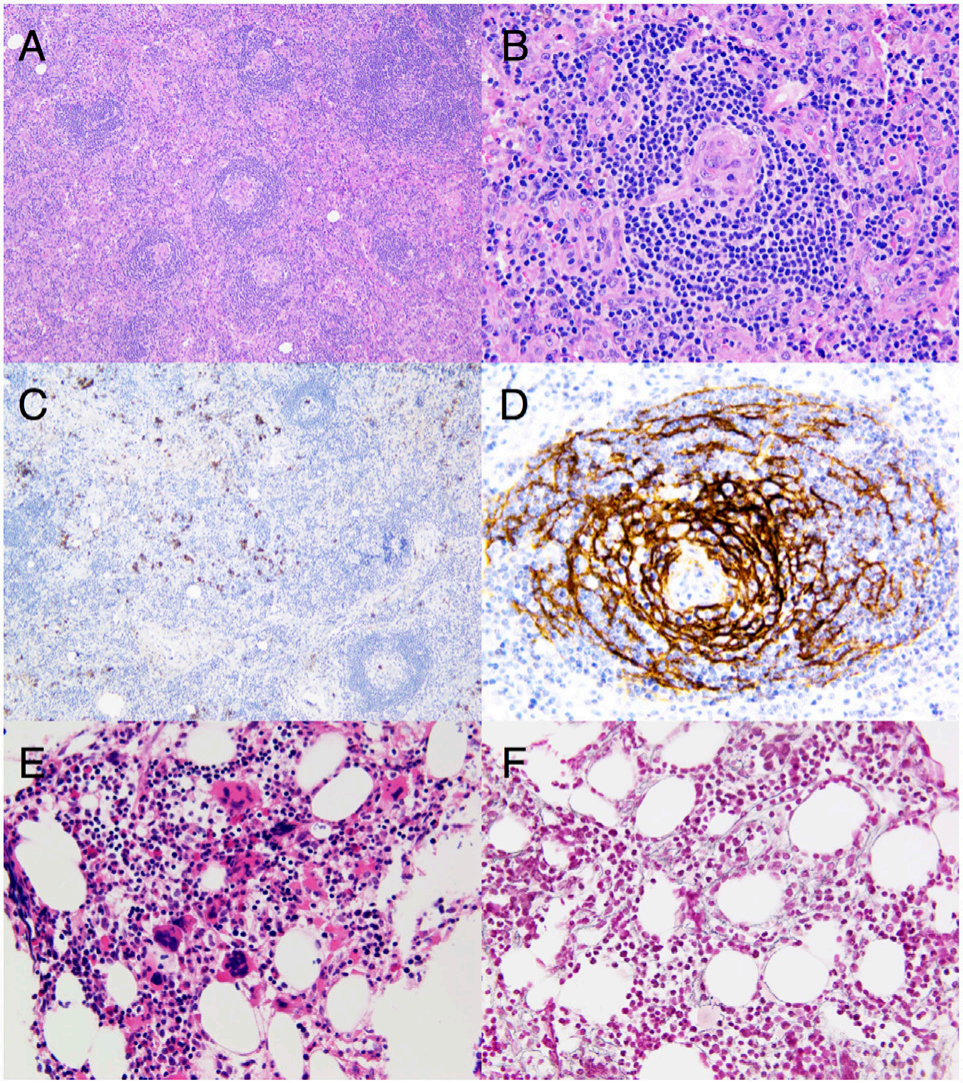
**(A)** Hematoxylin and eosin stain of a surgically removed axillary lymph node consistent with idiopathic multicentric Castleman’s disease (iMCD). Low magnification highlighting a prominent paracortex encircling atrophic secondary follicles (original magnification, ×10). **(B)** Hematoxylin and eosin stain focusing on an atrophic germinal center which is partially lymphocyte depleted and which is penetrated by a prominent, hyalinized blood vessel lined by plump endothelial cells with enlarged nuclei, giving the appearance of a lollipop. Note the marked vascular proliferation in the interfollicular areas (original magnification, ×40). **(C)** CD138 staining of sparsely scattered plasma cells in the interfollicular areas, less abundant compared to what is observed in the plasmacytic histopathological variant of iMCD (original magnification, ×10). **(D)** CD21 stain displaying a prominent network of follicular dendritic cells (FDCs) in an abnormal follicle with a concentric arrangement of the small lymphocytes of the mantle zone along with FDC nuclei (original magnification, ×40). **(E)** Hematoxylin and eosin stain of bone marrow showing megakaryocytic hyperplasia with a mixture of small hypolobated megakaryocytes and others having a multi-separated nucleus (original magnification, ×40). **(F)** Reticulin stain on the bone marrow trephine biopsy highlighting mild myelofibrosis (original magnification, ×40).

The etiology remained unclear and the patient underwent several supplementary investigations. An axillary lymph node was surgically removed and showed atreic secondary lymphoid follicles with hyalinized/vascular GCs. Interfollicular areas showed a marked vascular proliferation and contained a small number of CD138-positive plasma cells (Figures 1A–D). Immunohistochemistry for HHV-8 with latency-associated nuclear antigen-1 was negative. The histopathological findings were consistent with the newly defined diagnostic criteria for iMCD (11). The blood smear and the flow cytometry in the blood were normal without evidence for lymphoproliferative disease. A bone marrow trephine biopsy revealed megakaryocytic hyperplasia and mild reticulin myelofibrosis without abnormal lymphocytic infiltration (Figures 1E,F).

**Figure 2:**
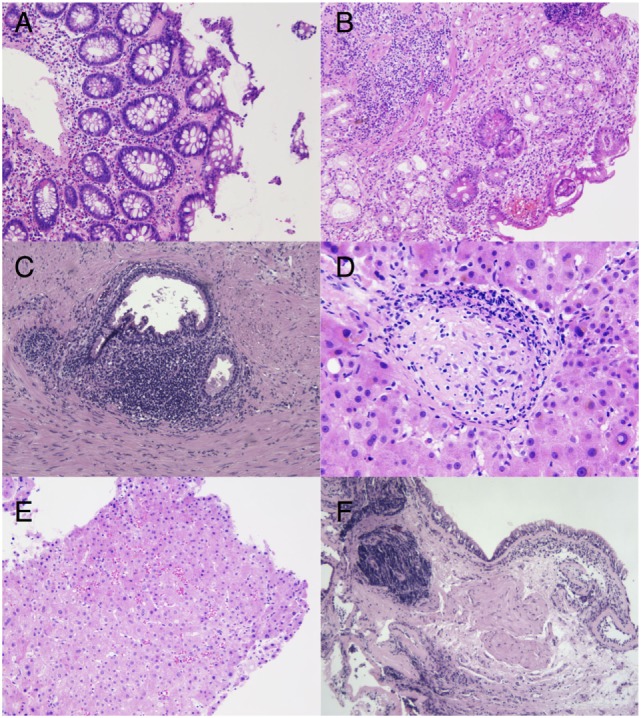
**(A)** Hematoxylin and eosin stain. Colon biopsy showing irregular thickening of the subepithelial basement membrane, encircling some capillaries, indicating a collagenous colitis (original magnification, ×20). **(B)** Hematoxylin and eosin stain. Glandular atrophy in the gastric body with foci of intestinal metaplasia and deep lymphoplasmacytic infiltrates, suggestive of an autoimune gastritis (original magnification, ×20). **(C)** Hematoxylin and eosin stain. Reactive periglandular chronic inflammation in the prostate (original magnification, ×10). **(D)** Hematoxylin and eosin stain. Rare lobular non-necrotizing epithelioid granulomas with peripheral lymphoid ring are found in addition to unspecific portal inflammation (original magnification, ×40). **(E)** Hematoxylin and eosin staining of a liver biopsy showing mild sinusoidal distension in the liver biopsy (original magnification, ×20). **(F)** Hematoxylin and eosin stain. Bronchial wall containing lymphoplasmocytic aggregates of variable densities with crush artifacts (original magnification, ×10).

Renal biopsy showed (1) thickening of the peripheral basement membrane and the intern lamina rara, (2) mesangial proliferation without glomerular deposition of immunoglobulin, and (3) a band-like pattern of interstitial fibrosis associated with lymphohistiocytic inflammatory cell infiltrates. Malignancies were also actively searched for. Colon biopsies showed a collagenous colitis rich in eosinophils (Figure 2A). Gastric biopsies revealed chronic inflammation and glandular atrophia with intestinal metaplasia of the fundus suggestive of an atrophic gastritis (Figure 2B). Prostatic biopsies showed mild chronic inflammation without neoplasia (Figure 2C). Liver biopsy showed mild sinusoidal distension, unspecific portal and lobular inflammation, and rare epithelioid granulomas without necrosis (Figures 2D,E). Finally, the patient underwent a bronchial biopsy showing unspecific chronic inflammation without granuloma, which was not suspicious for sarcoidosis (Figure 2F).

Together, these clinicopathological findings are compatible with a diagnosis of iMCD (11) and TAFRO syndrome (9).

### Treatment Course and Outcome

**Figure 3:**
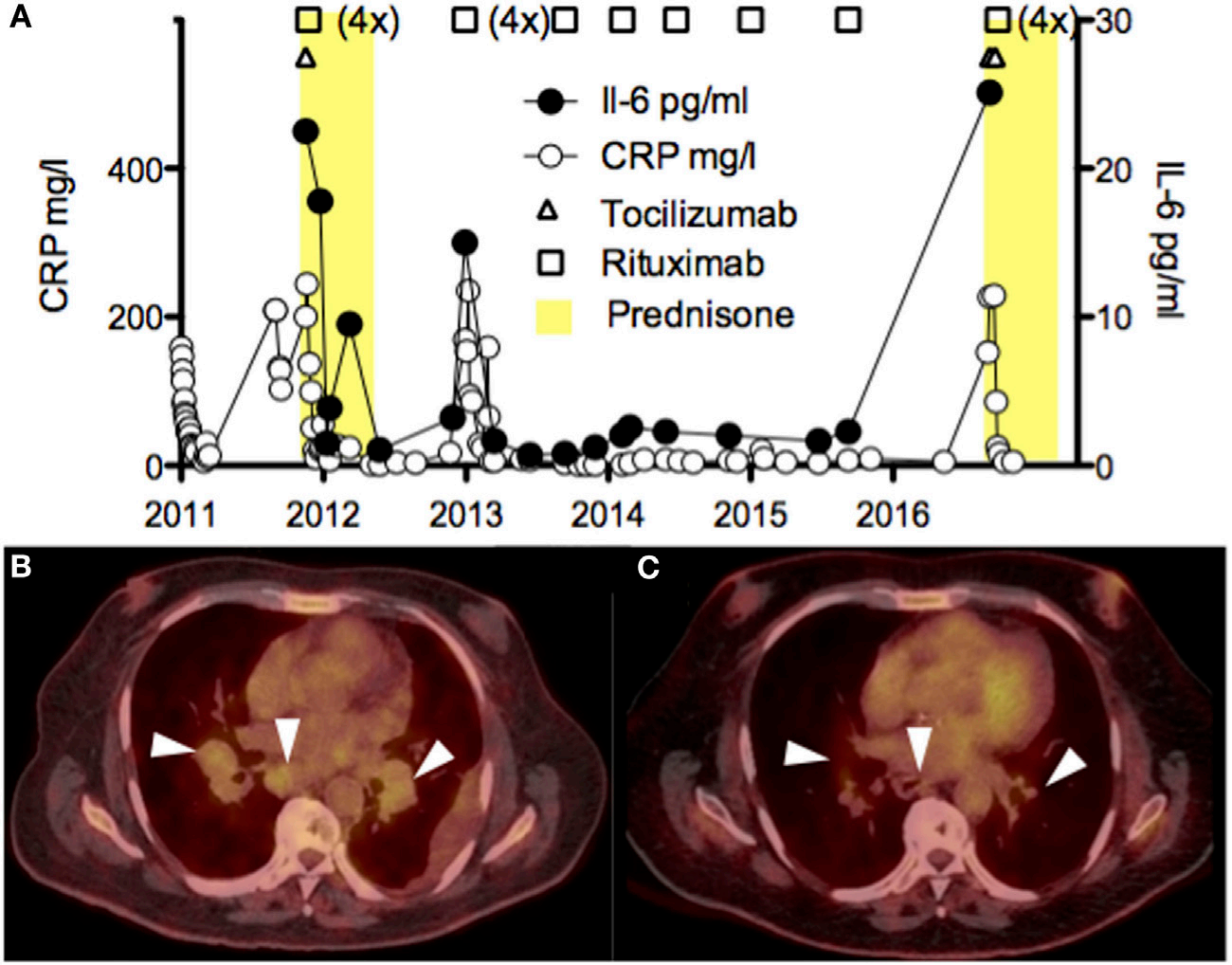
**(A)** Time course of the serum CRP (mg/l) and IL-6 (pg/ml) levels, and the different immunosuppressive drugs (corticosteroids, tocilizumab, and rituximab) administered to the patient. High values of IL-6 early after tocilizumab were excluded. **(B,C)** PET-CT scans showing pulmonary and mediastinal lymphadenopathies before **(B)** and after treatment with rituximab **(C)**.

The best management of iMCD disease remains poorly defined with only one randomized clinical trial published in 2014 on siltuximab compared to best supportive care (12). Thus, this case required multidisciplinary discussion and consensus in 2011 when the diagnosed was made and judgment was based on available data with tocilizumab (13) and rituximab for the treatment of HHV-8-associated MCD (14). Thus, treatment was initiated with high dose methylprednisolone (1 g/day for 3 days) transitioned to prednisone 1 mg/kg for several weeks, one single infusion of tocilizumab (8 mg/kg), and 1 week later rituximab 375 mg/m^2^ (total 4 weekly doses) (Figure 3A). The rational to use methylprednisolone and tocilizumab was to rapidly stop IL-6-mediated inflammation as induction regimen, whereas rituximab was used as maintenance treatment. We observed resolution of the inflammatory syndrome and slow clinical improvement over the course of 3 weeks, thrombocyte values normalized (>100 G/l) after 5 weeks and cholestasis parameters several months later. Corticosteroids were rapidly weaned off because of poor control of the diabetes, and the patient was hemodialyzed for end-stage renal disease. In the following 5 years, the patient relapsed twice, each time about 1 year after rituximab was discontinued (Figure 3A). The first relapse was successfully treated with rituximab alone and the second with methylprednisolone/tocilizumab (2 doses)/rituximab (4 weekly doses 375 mg/m^2^). Five years after the diagnosis was made (before the second relapse), the PET-CT showed reduction of the lymphadenopathies (Figures 3B,C). Auto-antibodies disappeared over the years and hypogammaglobulinemia developed, probably due to repeated rituximab treatments. Follow-up bone marrow biopsies were not performed in this case.

## Discussion

### TAFRO in Western Countries

TAFRO syndrome is a rare clinical syndrome of unknown origin that has been described in patients with iMCD. Most of the reported cases were Japanese patients. To analyze the role of ethnicity, we have reviewed the literature for all known Caucasian cases and have identified eight including the present one. When analyzing major and minor criteria recently established by Masaki et al., we confirmed that Caucasian cases were HIV/HHV-8-negative and meet both major and minor criteria (9, 15–20). In comparison with the Japanese cohort, of 18 patients we find Caucasian TAFRO cases presented more frequently with kidney failure and auto-immune antibodies, although these results need to be interpreted with caution due to the low number of patients (Table 1). Recently, a patient with TAFRO was also reported in Latin America (21) and the members of the Castleman Disease Collaborative Network report treating iMCD patients with TAFRO from diverse ethnic backgrounds throughout the world for decades (11). As to IgG4-related disease primary reported in Japan (22), TAFRO syndrome is a rare entity affecting patients with different ethnic background without major differences in the clinicopathological presentation.

**Table 1:** Clinical characteristics and laboratory data of eight Caucasian patients with TAFRO compared to the Japanese cohort.

	Patient 1 Abdo et al. (19)	Patient 2 Tedesco et al. (17)	Patient 3 Allegra et al. (18)	Patient 4 Jouvray et al. (15)	Patient 5 Simons et al. (16)	Patient 6 Iwaki et al. (20)	Patient 7 (unpublished)	Patient 8 (present case)	Summary	Japanese Masaki et al. (9)
Origin	France	Italy	Italy	France	USA	USA	USA	Portugal	8	18
Genre/age	M/81	F/21	M/66	F/32	M/22	M/25	F/35	M/67	M: (5/8) mean 62.5	M: (8/18) mean 44.4
Anasarca (pleural effusion, ascites, general edema)	Yes	Yes	Yes	Yes	Yes	Yes	Yes	Yes	100% (8/8)	100% (18/18)
Thrombocytopenia (<100,000/μl)	Yes	Yes	Yes	Yes	Yes	Yes	Yes	Yes	100% (8/8)	100% (18/18)
Systemic inflammation (fever unknown etiology >37.5 and/or CRP >2 mg/dl)	Yes	Yes	Yes	Yes	Yes	Yes	Yes	Yes	100% (8/8)	83% (15/18)
Castleman’s disease-like features on lymph node biopsy	Mixed type CD	Mixed type CD	No data	Mixed type CD	Mixed type CD	Mixed type CD	Mixed type CD	Mixed type hyaline vascular	100% (8/8)	92% (12/13)
Reticulin myelofibrosis and/or increased number of megakaryocytes in bone marrow	Yes	Yes	Yes	No data	Yes	Yes	Yes	Yes	100% (7/7)	75% (9/12)
Mild organomegaly (hepatomegaly, splenomegaly and lymphadenopathy)	Yes	Yes	Yes	Yes	Yes	Yes	Yes	Yes	100% (8/8)	94% (17/18)
Progressive renal insufficiency	Yes	Yes	Yes	Yes	Yes	Yes	Yes	Yes	100% (8/8)	55% (10/18)
Human herpesvirus 8/HIV	Negative 2×	Negative 2×	No data	Negative 2×	No data	Negative 2×	Negative 2×	Negative 2×	100% (8/8)	100% (18/18)
IL-6	Elevated	Elevated	No data	No data	Elevated	Normal	Elevated	Elevated	88% (7/8)	66% (8/12)
VEGF	Normal	No data	No data	No data	No data	Elevated	Elevated	Elevated	75% (3/4)	62% 5/8
Immunity anomalies	Anti-SSA/anti SSB	ANA	Anti-cardiolipin	Anti TPO	No data	Anti-SSA/ANA	No data	Anti-SSA/ANA	100% (6/6)	61% (11/18)
Complement anomalies	No data	Normal	No data	C4 decreased	No data	C4 decreased	C4 decreased	Normal	67% (3/4)	–
Immunoglobulin level	Elevated (polyclonal)	Elevated (polyclonal)	Normal	Decreased	No data	Normal	Normal	Normal	Normal/low 71% (5/7)	Normal/low 69% (11/16)
LDH	No data	Elevated	Elevated	Elevated	No data	Elevated	Normal	Elevated	Decreased 0% (0/6)	Decreased 25% (4/16)
Alkaline phosphatase	Normal	No data	Elevated	Elevated	No data	Elevated	Elevated	Elevated	87% (6/7)	64% (9/14)
Thrombotic microangiopathy	No data	No data	No data	Yes (confirmed by renal biopsy)	No data	No data	No data	Possible (renal biopsy)	50% (1/2)	–
Albumin	Low	Low	Low	Low	No data	No data	Low	Low	100% (6/6)	–
EBV viremia	No data	Negative (PCR)	Negative (PCR)	Negative (PCR)	DNA positive in the lymph node and bone marrow	Negative (PCR)	Negative	Positive (PCR)	33% (2/6)	–
CMV activation	No data	Negative (serology)	Negative (PCR)	Negative (PCR)	No data	Negative (PCR)	Negative	Negative (PCR)	0% (0/5)	–
Treatment	Prednisone	Prednisone, tocilizumab then R-CHOP	Prednisone and ciclosporin A	Prednisone and plasmapheresis then tocilizumab then anakinra	Tocilizumab then rituximab and etoposide	Prednisone, then rituximab, then siltuximab, then VDT-ACE-R (3×), then ciclosporin A and IVIg	Methylprednisolone and rituximab	Prednisone and rituximab then tocilizumab	–	–
Follow-up	1 year	1 year	8 months	9 years	2 years	6.75 years	4 years	6 years	–	–
Outcome	Survival	Survival	Death	Survival (relapse)	Survival	Survival (multiple relapse)	Survival	Survival (multiple relapse)	Death 13% (1/8)	Death 11% (2/18)

*VDT-ACE-R, velcade–dexamethasone–thalidomide–adriamycin–cytoxan–etoposide–rituximab; CHOP, cyclophosphamide–hydroxyadriamycine–oncovin–prednisone*.

### TAFRO: A Spectrum of Castleman’s Disease?

**Figure 4:**
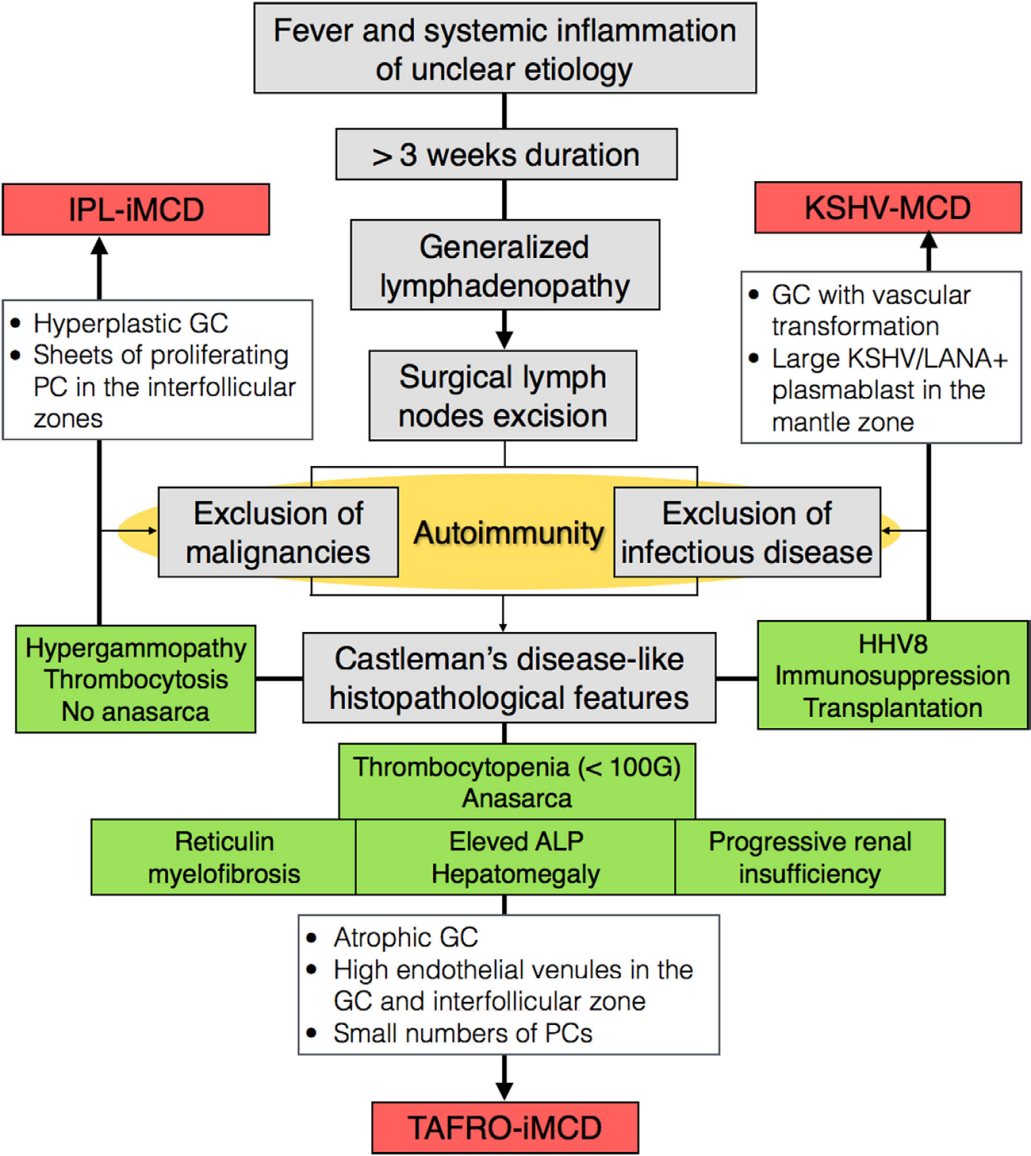
Classification algorithm for TAFRO-iMCD, IPL-iMCD, and KSVH-MCD. Abbreviations: iMCD, idiopathic multicentric Castleman disease; MCD, multicentric Castleman disease; HV, hyaline vascular; IPL, idiopathic plasmacytic lymphoadenpathy; KSHV, Kaposi sarcoma-associated herpesvirus; GC, germinal center; ALP, alkaline phosphatase; HHV-8, human herpesvirus 8; PC, plasma cell; LANA, latency-associated nuclear antigen.

Recently, the international working group for the Castleman Disease Collaborative Network established the first-ever diagnostic criteria for HHV-8-negative-iMCD analyzing 244 patients with iMCD (128 patients from literature, 37 cases submitted by the working group members, and 79 from randomized control study) and more specifically the histopathological features of 85 lymph nodes with presumptive diagnosis of MCD. Importantly, this cohort included TAFRO and non-TAFRO-iMCD cases (11, 23). The authors argued that while there are clinical differences between iMCD patients with TAFRO syndrome and those who do not have TAFRO syndrome, there are a significant number of overlapping histopathological and clinical features. Furthermore, some patients with TAFRO clinical syndrome did not present with the more classical HV histopathology, and some patients with the more classical histopathology for TAFRO syndrome did not have TAFRO clinical features. There was consensus among the international working group that TAFRO syndrome is a clinical subtype of iMCD to be included in the diagnostic criteria, not a separate entity. Nevertheless, a consensus between both the international working group on iMCD and the Japanese working group on TAFRO is warranted to agree on criteria, prevalence, and incidence of TAFRO. We proposed a classification algorithm for MCD (Figure 4). An exhaustive list of major, minor, and exclusion criteria can been found elsewhere (11). More importantly, genomic, transcriptomic, and proteomic studies are needed to understand molecular differences that may exist. The clinical utility of subtyping the lymph node histological features into HV, plasmacytic, or mixed type also warrants further investigation.

### TAFRO and Auto-Immunity

The relationship between TAFRO/iMCD and auto-immunity is poorly understood. In our study, auto-antibodies such as anti-nuclear or anti-SS-A (SS-A) were found in all six patients with available data (Table 1). Albeit auto-antibodies alone are insufficient to make a definite diagnosis of connective tissue disease such as systemic lupus erythematosus (SLE), Sjögren syndrome, systemic sclerosis, or dermatomyositis/polymyositis, the classification criteria to diagnose undifferentiated connective tissue disease can be met (24). Furthermore, generalized lymphadenopathies are a common finding in about a quarter of patients with SLE (25, 26). When analyzing the histopathological findings in lymph node biopsies from 19 patients with active SLE, iMCD-compatible lesions were present in 5; 3 of mixed type and 2 of HV type (27). As to anti-SS-A52 auto-antibodies, they seem to be more prevalent in patients with TAFRO as illustrated by this study and reported by others (19, 20, 28). Interestingly, undiagnosed anti-SS-A 52 positive patients do not frequently develop definitive connective tissue disease (29). Finally, the chemokine CXCL10 (IP-10) involved in the pathogenesis of many auto-immune disease including Sjögren syndrome (30) seems to be increased during the flares-up in TAFRO (31). In conclusion, these data suggest that auto-immunity is an important aspect of TAFRO.

### TAFRO and Kidney Disease

Kidney involvement is frequently observed in patients with TAFRO syndrome, the underlying mechanisms, however, are poorly understood. Depending on the authors, the R in TAFRO may refer to renal dysfunction or reticulin myelofibrosis (9, 20). Iwaki et al.’s recently proposed diagnostic criteria for TAFRO that do not include renal failure as major or minor criteria (20). Importantly, all Caucasian TAFRO patients who we have found had renal failure (Table 1). The clinical presentation of iMCD patients with renal failure is frequently associated with arterial hypertension, glomerular hematuria, and proteinuria. The most frequent lesions were endotheliosis of the small-vessel and glomerular double contours with glomerular/arteriolar thrombi, a pattern similar to what can be found in thrombotic microangioapthy (TMA) (32, 33). Of interest, 2/7 patients with TAFRO had renal insufficiency and lesions compatible with MAT. Furthermore, complement, an important trigger for MAT (34), can also be frequently decreased in TAFRO (Table 1). Thus, these results stress the importance of regularly monitoring kidney function in patients with TAFRO syndrome particularly in the presence of other risk factors such as diabetes as was the case for our patient. Further studies into the link between TAFRO, complement dysregulation, and MAT are needed.

### Conclusion

TAFRO syndrome is a rare subtype of iMCD resulting in generalized organ inflammation of unknown origin. Diagnosis remains very challenging and the exclusion of infectious, auto-immune, and neoplastic disorders is necessary. Patients undergo a series of clinical investigations and biopsies, which are often non-contributive. In this article, we demonstrate a Caucasian case of TAFRO syndrome and lend further support to the notion that TAFRO syndrome can be seen in iMCD patients around the world. Genomic, transcriptomic, and proteomic investigations into the etiology, pathogenesis, relationship between auto-immunity, complement dysregulation, and kidney failure, and molecular differences between TAFRO-iMCD and non-TAFRO-iMCD are warranted.

## Consent

Written informed consent was obtained from the patient for publication of this Case report and any accompanying images. A copy of the written consent is available for review by the Editor of this journal.

## Ethics Statement

Written informed consent was obtained from the patient for publication of this Case report and any accompanying images as per our standard institutional rules. A copy of the written consent is available for review by the Editor of this journal.

## Author Contributions

Conceived and designed the experiments: YM, CL, and SV. Analyzed the data: YM, CL, SV, DF, KS, JS, and YC. Contributed reagents/materials/analysis tools KS, YC, LT, DL, DF, JS, and SV. Critical discussion and reading: YM, JS, DF, KS, YC, LT, and DL. Wrote the paper: YM, CL, and DF.

## Conflict of Interest Statement

The authors declare that the research was conducted in the absence of any commercial or financial relationships that could be construed as a potential conflict of interest.

## Abbreviations

iMCD, idiopathic multicentric Castleman’s disease; SLE, systemic lupus erythematosus; IPL, idiopathic plasmacytic lymphadenopathy; HHV-8, human herpesvirus 8.
